# Tumor mutation burden (TMB)-associated signature constructed to predict survival of lung squamous cell carcinoma patients

**DOI:** 10.1038/s41598-021-88694-7

**Published:** 2021-04-27

**Authors:** Dan Yan, Yi Chen

**Affiliations:** 1 Department of Respiratory, Jinhua Municipal Central Hospital, Jinhua Hospital of Zhejiang University, No. 365, East Renmin Road, Jinhua, 321000 Zhejiang Province People’s Republic of China; 2 Department of Hepatology, The First Affiliated Hospital of Wenzhou Medical University, Wenzhou, 325000 People’s Republic of China

**Keywords:** Cancer, Genetics

## Abstract

Lung squamous cell carcinoma (LUSC) is a common type of lung cancer with high incidence and mortality rate. Tumor mutational burden (TMB) is an emerging biomarker for selecting patients with non-small cell lung cancer (NSCLC) for immunotherapy. This study aimed to reveal TMB involved in the mechanisms of LUSC and develop a model to predict the overall survival of LUSC patients. The information of patients with LUSC were obtained from the cancer genome atlas database (TCGA). Differentially expressed genes (DEGs) between low- and the high-TMB groups were identified and taken as nodes for the protein–protein interaction (PPI) network construction. Gene oncology (GO) enrichment analysis and gene set enrichment analysis (GSEA) were used to investigate the potential molecular mechanism. Then, we identified the factors affecting the prognosis of LUSC through cox analysis, and developed a risk score signature. Kaplan–Meier method was conducted to analyze the difference in survival between the high- and low-risk groups. We constructed a nomogram based on the risk score model and clinical characteristics to predict the overall survival of patients with LUSC. Finally, the signature and nomogram were further validated by using the gene expression data downloaded from the Gene Expression Omnibus (GEO) database. 30 DEGs between high- and low-TMB groups were identified. PPI analysis identified CD22, TLR10, PIGR and SELE as the hub genes. Cox analysis indicated that FAM107A, IGLL1, SELE and T stage were independent prognostic factors of LUSC. Low-risk scores group lived longer than that of patients with high-risk scores in LUSC. Finally, we built a nomogram that integrated the clinical characteristics (TMN stage, age, gender) with the three-gene signature to predict the survival probability of LUSC patients. Further verification in the GEO dataset. TMB might contribute to the pathogenesis of LUSC. TMB-associated genes can be used to develope a model to predict the OS of lung squamous cell carcinoma patients.

## Introduction

Lung cancer is the commonest cancer, and is the main cause of global tumor morbidity and mortality^1^. Non-small cell lung cancer (NSCLC) is a common type of lung cancer, including lung adenocarcinoma (LUAD), lung squamous cell carcinoma (LUSC) and large cell lung carcinoma (LCLC). Among NSCLC, LUAD is the most common followed by LUSC which constitutes approximately 30% of all lung cancer cases^2^. With the discovery of target genes and the development of targeted therapy and immunotherapy, the survival of patients with NSCLC has been extended^3^, especially patients with LUAD^4^. However, due to the lack of effective targeted treatments, the progress of LUSC therapy is very slow and the clinical outcome of LUSC remains unsatisfactory.

TMB is the number of mutations per million bases in tumor tissue, including base substitutions, gene insertion, and gene coding and deletion errors. TMB has a vital role in tumor occurrence and development, and affects the immune response and survival prognosis of LUSC patients^5^. And many studies suggest that TMB is a potential and emerging biomarker for selecting NSCLC patients suitable for immunotherapy, even better than PD-1/PDL-1 expression^6,7^. In resected NSCLC patients, decreased TMB is believed to be associated with poor prognosis ^8^.

NSCLC patient prognosis is most often evaluated in light of the American Joint Committee on Cancer (AJCC) staging system (8th edition), with the stage of the cancer being used to guide treatment decision making^9^. However, in many cases this system may fail to accurately predict the prognosis of a given patient, as a number of other factors can influence such outcomes. Several studies have suggested that TMB might be a potentially-useful clinical predictor in NSCLC patients undergoing immunotherapy^10^ and patients with resected NSCLC^8^. However, there are few studies focusing on the relationship between TMB and LUSC. However, few studies have focused on the relationship between TMB and LUSC. Therefore, using data from the TCGA database, we built a novel predictive model able to predict the survival probability of LUSC patients.

## Method

### Data acquisition

The RNA transcriptome sequences and corresponding clinicopathological data for LUSC patients were collected from The Cancer Genome Atlas (TCGA, https://portal.gdc.cancer.gov/) and used for model training; this included 502 tumors and 49 paracancerous tissues. Information obtained from the Gene Expression Omnibus (GEO) database (https://www.ncbi.nlm.nih.gov/geo/) was used for external validation; this included 69 lung squamous carcinoma samples (GSE73403). Data was obtained solely from public databases, obviating any ethical conflict.

### TMB calculation and differential expressed genes (DEGs) screening

TMB is the sum of mutations per megabase in tumor tissue. TMB for each organization can be detected using the VarScan method, as calculated by the R package "maftools". The R package "limma" was used to identify differentially expressed genes (DEGs) between a high TMB population and a low TMB population. Classification used the median of the TMB score and a |log2 fold change |≥ 1.0. A *P* value < 0.05 and a False Discovery Rate (FDR) < 0.05 were the screening criteria. TMB evaluation of different analysis pipelines (MuTect and Muse detected Mutation data) was undertaken using R package ‘maftools’. Kaplan–Meier analysis was used to show the survival difference between high and low TMB expression groups.

### Protein–protein interactions (PPI) network construction

We construction a PPI network of DEGs using the STRING database (https://string-db.org/) and visualized using Cytoscape software. A confidence score of C ≥ 0.15 was defined as the threshold criterion.

### Functional enrichment analysis

We used Gene Set Enrichment Analysis (GSEA) to analyze the signal path for two different TMB expression groups. Gene Ontology (GO) enrichment analysis was used to reveal potential biological processes of TMB-associated DEGs in LUSC.

### Construct and validate a Cox proportional hazards model

Univariate and multivariate Cox analysis were used to identify factors affecting survival and prognosis of LUSC. The risk associated with TMB-related genes was found from multivariate Cox regression analysis. From the median calculated score, patients were classified into high- and low-risk groups. Kaplan–Meier curves showed the survival difference between groups for both training and validation groups. A nomogram utilizing the risk score and baseline clinical information was able to predict 1-, 3- and 5-year overall survival (OS) in LUSC patients. The predictive accuracy of the model was found using ROC curves. RNA transcriptome profiles of LUSC patients were downloaded from the GEO database and used for external validation.

### Statistical analysis

DEGs were screened using the R package "Limma". GO was conducted using “clusterProfiler”, “ggplot2”, “enrichplot”, “stringi”, and “DOSE” packages. By using the TMB value, GSEA was able to analyze the signal path. We choose the "c2.cp.kegg.v6.2.symbols.gmt” gene set downloaded from the MSigDB database (http://software.broadinstitute.org/gsea/msigdb/) as the reference. To assess prognostic value, Cox regression analysis was used to estimate the hazard ratio and 95% confidence interval (95% CI) for each variable in the LUSC cohort. Statistically significant variables (*P* < 0.05) in the initial univariate Cox regression analysis were used in the subsequent multivariate analysis. A predictive risk model was created using multivariate Cox regression analysis implemented in the “survival” package. The resulting model and clinical information were incorporated into a final predictive nomogram, able to predict 1-, 3-year and 5-year LUSC patient OS. Nomogram prediction was undertaken using the “survival” package. Predictive accuracy was evaluated using ROC curves. All statistical analysis was performed using R (version 3.5.2), Cytoscape, GSEA, and perl. A *P* value < 0.05 was considered significant.

## Result

### DEGs screening and PPI network construction

**Figure 1:**
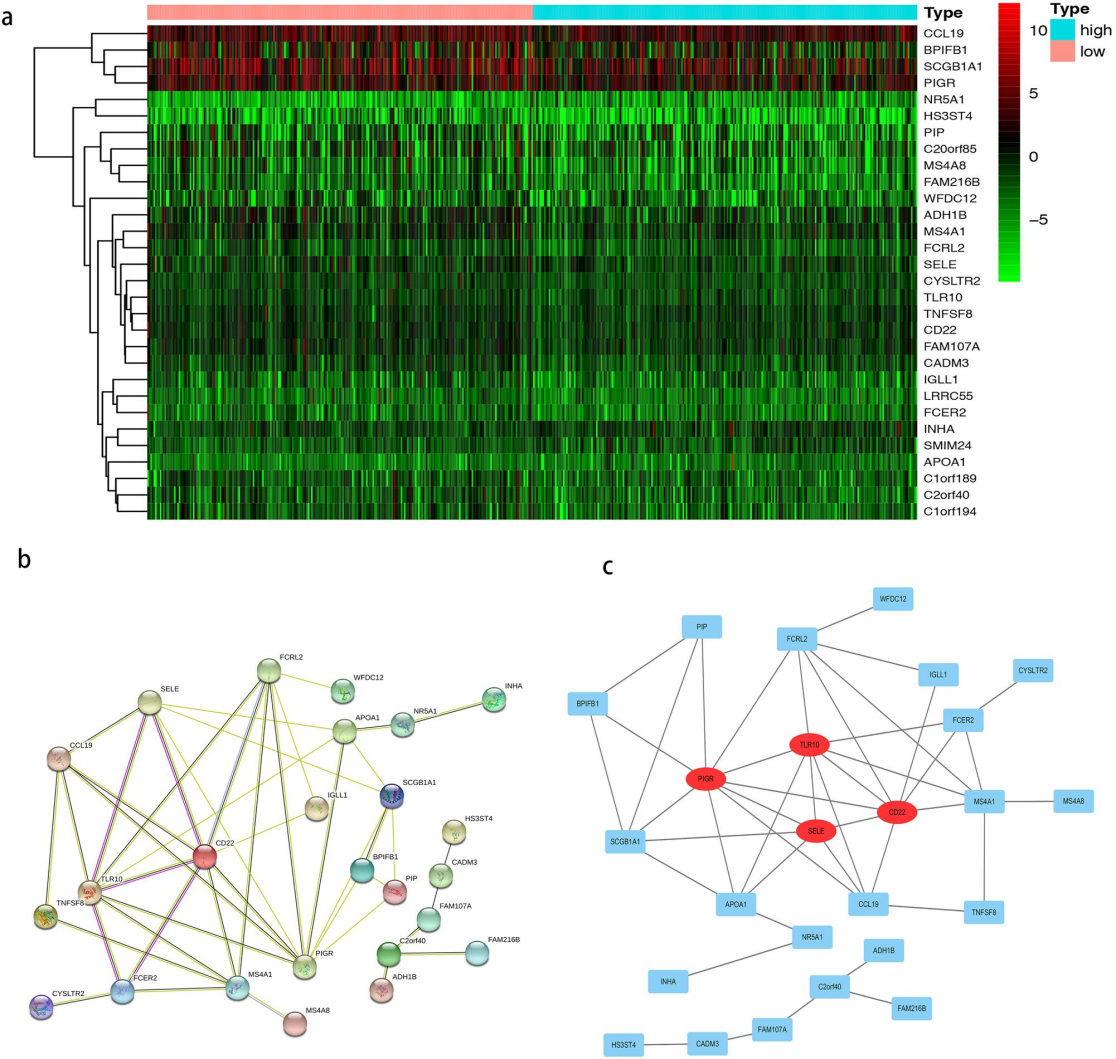
Identification of DEGs in LUSC between tumor and normal tissues. (**a**) The heatmap of DEGs between the high‐TMB and low‐TMB groups in LUSC by analysis of the TCGA datasets. Each column represents a sample, and each row represents one of DEGs. The levels of DEGs are shown in different colors, which transition from green to red with increasing proportions. The lines before the heat map indicated the dendrogram of DEGs cluster analysis. (**b**) The protein–protein interaction network (PPI) analysis was constructed by all the 30 DEGs using STRING database. (**c**) Four hub genes (PIGR, TLR10, SELE and CD22) in the PPI were screened by Cytoscape based on their connectivity degree. Red circles indicated four hub genes.

**Figure 2:**
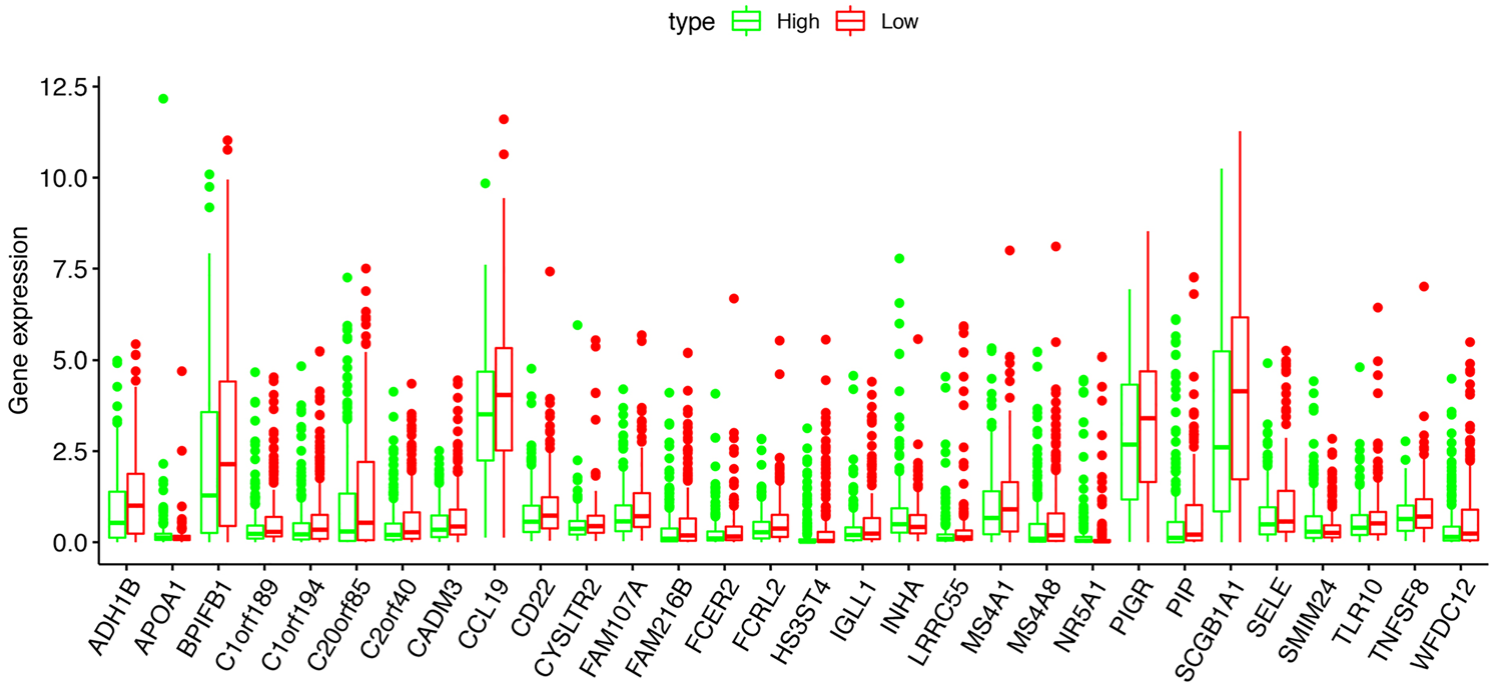
The expression of DEGs distributed in high-TMB and low-TMB groups.

30 DEGs (including CCL19, BPIFB1, SCGB1A1, PIGR, SELE, NR5A1, PIP) were sorted into low and high expression TMB groups: threshold |Log2 FC|> 1.0, P value < 0.05 and FDR < 0.05 (in Table1; Fig. 1a). Kaplan–Meier analysis indicated patient OS in the low expression group was significantly lower than in the high expression group. When evaluating TMBs generated by different analysis pipelines this remained true (Fig. 5). Figure 2 shows the distribution of DEGs in the two TMB expression groups. By using a STRING database comprising 25 nodes and 44 edges, we established a PPI network between DEGs, using a confidence threshold of C ≥ 0.15 (Fig. 1b). CD22, TLR10, PIGR, and SELE were identified as hub genes and visualized using Cytoscape (https://cytoscape.org/) (Fig. 1c). DEGs included in the model and hub genes are verified in TMB evaluation of different analysis pipelines (Table 2).

**Table 1:** Differential expressed genes between low TMB and high TMB groups.

Gene	logFC	pValue
CYSLTR2	− 1.6898991	0.00010269
MS4A1	− 1.1441338	0.00058734
FAM107A	− 1.0200621	7.73E−05
IGLL1	− 1.7638948	0.00533523
LRRC55	− 1.785876	0.00055621
MS4A8	− 1.8295919	0.00021727
C20orf85	− 1.1821732	0.00062144
SELE	− 1.2084087	0.00472061
TNFSF8	− 1.1427382	0.0032476
NR5A1	1.9226092	7.87E−06
CADM3	− 1.2047907	5.53E−05
FCRL2	− 1.159438	0.0008174
BPIFB1	− 1.4510685	0.00011556
ADH1B	− 1.1416372	9.09E−06
INHA	1.85340517	0.00362178
SCGB1A1	− 1.1115467	1.63E−05
PIGR	− 1.0412167	0.00022015
C1orf189	− 1.1341047	0.00026482
WFDC12	− 1.2491682	0.00164705
FAM216B	− 1.3900118	2.55E−06
HS3ST4	− 1.504201	0.00026443
PIP	− 1.0171485	0.00026387
CD22	− 1.0772369	0.00013723
FCER2	− 1.3194606	0.00204346
C2orf40	− 1.2412141	3.72E−05
CCL19	− 1.073972	0.00058595
TLR10	− 1.0166526	0.00302647
C1orf194	− 1.0624677	0.00026991
APOA1	6.52638412	0.00622402
SMIM24	1.10145976	0.00793992

**Table 2:** DEGs are verified in TMB evaluation of different analysis pipelines.

Gene	SomaticSniper		MuTect		Muse	
	logFC	pValue	logFC	pValue	logFC	pValue
CD22	− 0.738376	1.22E−09	− 0.8605324	0.00718201	− 0.4142631	0.00088519
SELE	− 1.3754053	7.29E−05	− 1.0018508	0.01206434	− 0.5736792	0.00823645
PIGR	− 1.1479969	1.06E−06	− 0.7435242	0.00391641	− 1.0187546	0.00018397
TLR10	− 1.0942878	1.68E−07	− 0.9334027	0.03117443	− 0.9074117	0.01194325
FAM107A	− 1.2206673	1.58E−07	− 0.6545071	0.00402084	− 0.860165	0.00012132
IGLL1	− 2.1140051	2.21E−07	− 0.9500279	0.02153361	− 1.5941	0.01274359

### Enrichment analysis

**Figure 3:**
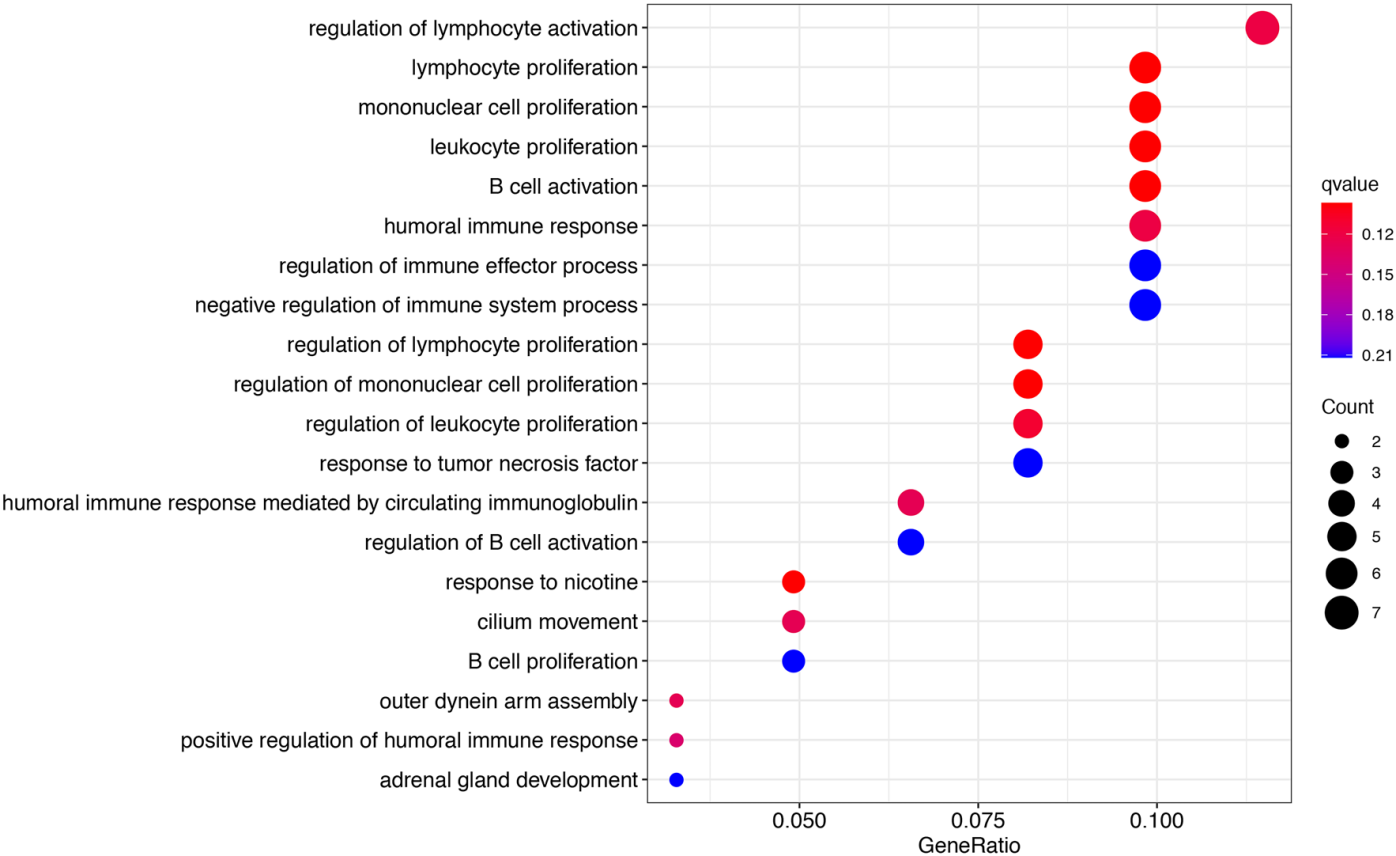
GO enrichment analysis of the DEGs between high- and low-TMB groups.

**Figure 4:**
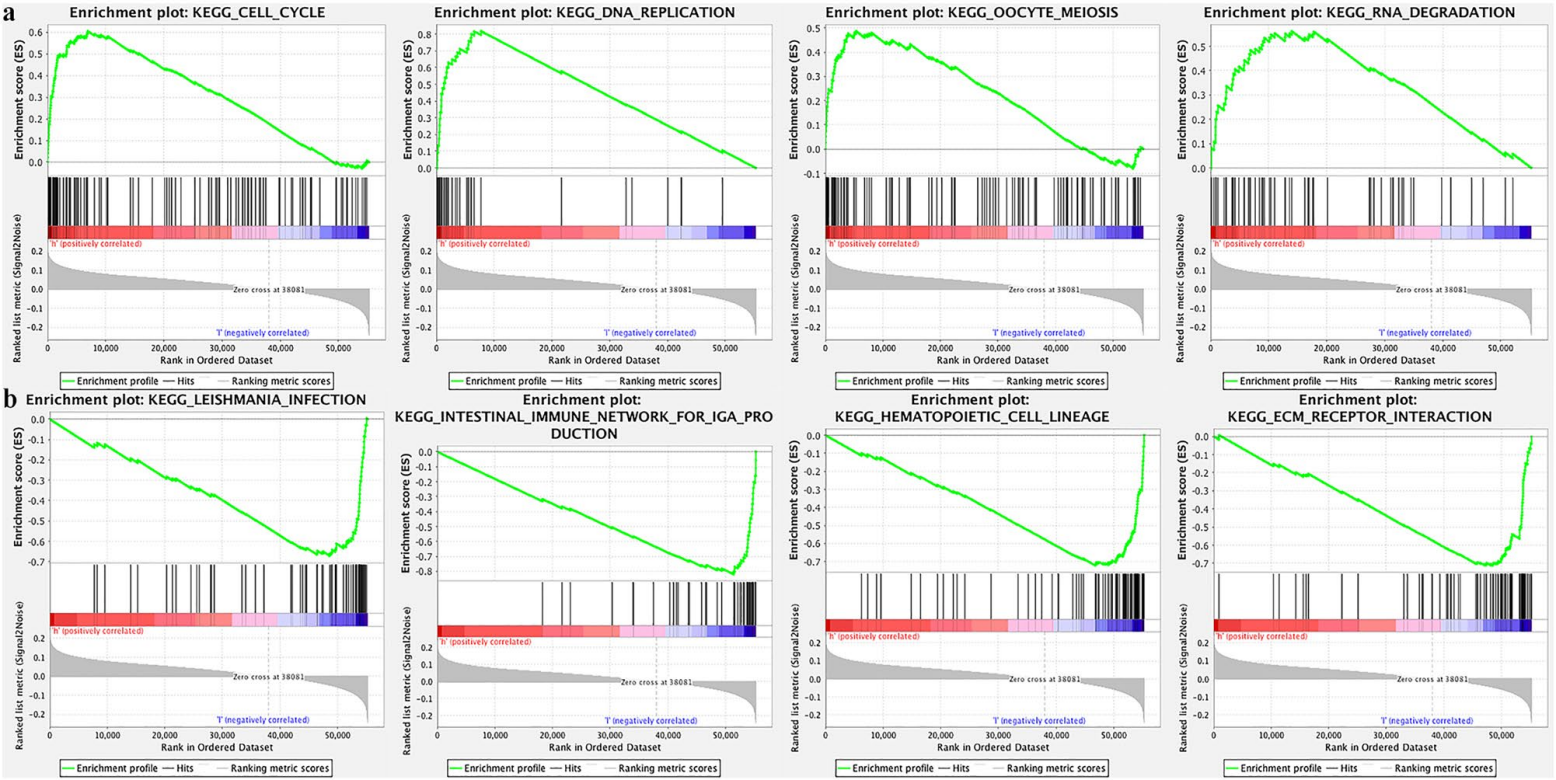
GSEA analysis was performed to further screen the significant pathway between high TMB group and low TMB group. The q‐value < 0.05 was considered as significance. (**a**) Significant pathway identified in the high-TMB group. (**b**) Significant pathway identified in the low-TMB group.

GO analysis indicated that the identified DEGs participated in regulation of lymphocyte activation, lymphocyte, mononuclear cell, and leukocyte proliferation (Table 3; Fig. 3). MS4A1, TNFSF8, SCGB1A1, CD22, and CCL19 were associated with lymphocyte, Leukocyte and mononuclear cell proliferation. TNFSF8, SCGB1A1, CD22, and CCL19 were involved with regulating lymphocyte proliferation and leukocyte proliferation. GSEA analysis indicated that high expression of TMB correlated with DNA replication, cell cycle, oocyte meiosis, spliceosome, RNA degradation, base excision repair, and pyrimidine metabolism. Low-TMB was mainly associated with B cell receptor, T cell receptor, and chemokine signaling pathways (Fig. 4).

**Table 3:** GO enrichment analysis of TMB associated DEGs in LUSC.

Description	*P* value	geneID
Lymphocyte proliferation	0.007	MS4A1/TNFSF8/SCGB1A1/CD22/CCL19
Mononuclear cell proliferation	0.007	MS4A1/TNFSF8/SCGB1A1/CD22/CCL19
Regulation of lymphocyte activation	0.007	IGLL1/TNFSF8/INHA/SCGB1A1/CD22/CCL19
Leukocyte proliferation	0.007	MS4A1/TNFSF8/SCGB1A1/CD22/CCL19
Regulation of lymphocyte proliferation	0.018	TNFSF8/SCGB1A1/CD22/CCL19
Regulation of mononuclear cell proliferation	0.018	TNFSF8/SCGB1A1/CD22/CCL19
Regulation of leukocyte proliferation	0.020	TNFSF8/SCGB1A1/CD22/CCL19
Negative regulation of immune system process	0.026	BPIFB1/INHA/SCGB1A1/CD22/APOA1
Regulation of endocytosis	0.037	SELE/CD22/CCL19/APOA1
adrenal gland development	0.037	NR5A1/APOA1
B cell receptor signaling pathway	0.038	MS4A1/IGLL1/CD22
B cell activation	0.038	MS4A1/IGLL1/INHA/CD22
Response to tumor necrosis factor	0.038	SELE/TNFSF8/CCL19/APOA1
Negative regulation of production of molecular Mediator of immune response	0.041	CD22/APOA1
negative regulation of Interferon-gamma production	0.041	INHA/SCGB1A1
positive regulation of Endocytosis	0.041	SELE/CCL19/APOA1
Mucosal immune response	0.041	BPIFB1/PIGR
Lymphocyte differentiation	0.041	MS4A1/TNFSF8/INHA/CCL19
Regulation of T cell proliferation	0.041	TNFSF8/SCGB1A1/CCL19

### Cox proportional hazards model construction

**Figure 5:**
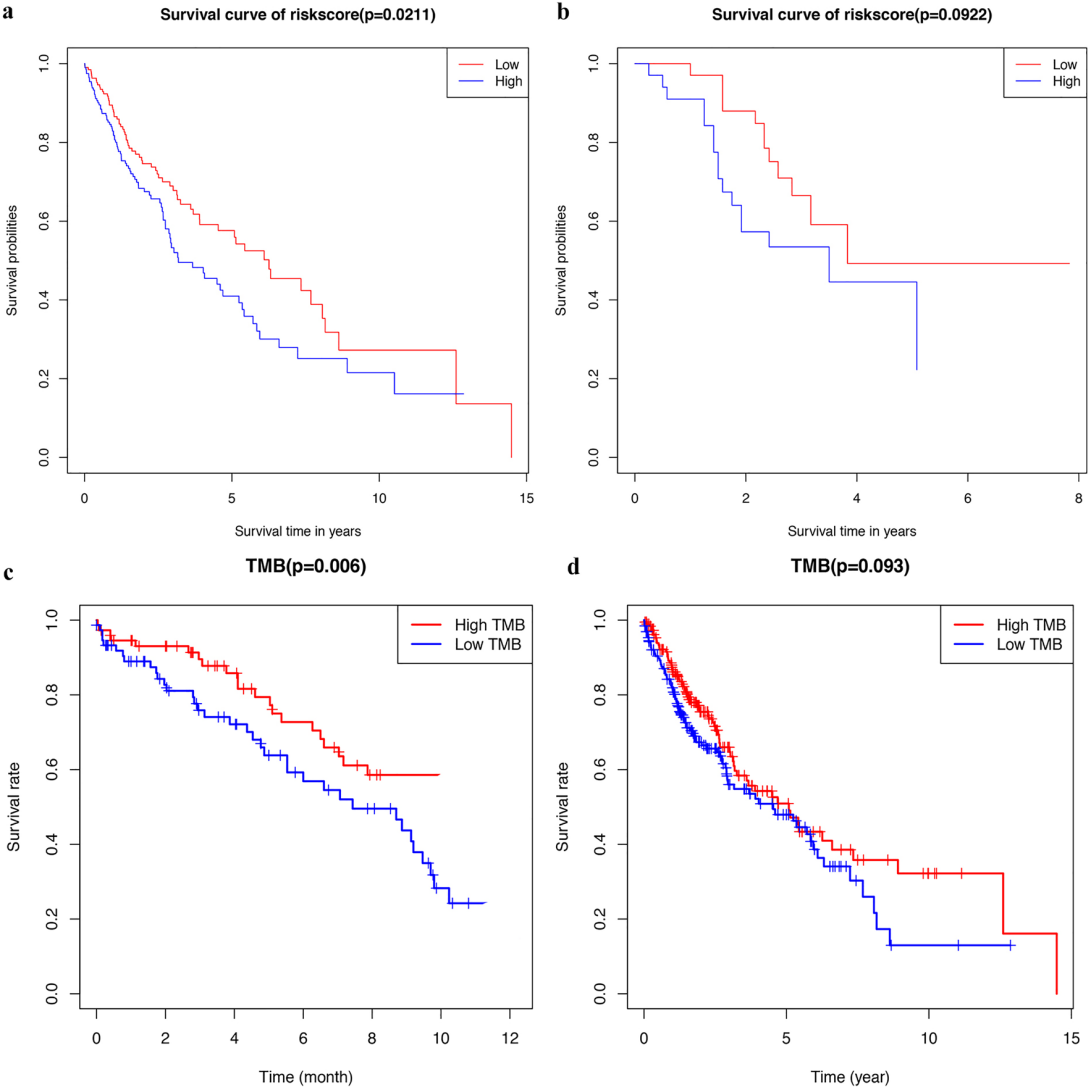
Kaplan–Meier survival curves. (**a**/**b**) Patients from the TCGA and GSE73403 dataset are stratified into two groups according to median values for the risk scores calculated by three gene based on risk score signature. (**a**) Kaplan–Meier survival curves of the signature in TCGA dataset. (**b**) Kaplan–Meier survival curves of the signature in GSE73403 dataset. (**c**) Kaplan–Meier survival curves of different TMB groups calculated by VarScan. (**d**) Kaplan–Meier survival curves of different TMB groups calculated by MuTect. (Red means high-TMB group and blue means low-TMB group).

Univariant analysis suggested that survival rate was correlated with FAM107A, IGLL1, SELE and T stage. Multivariate Cox analysis indicated that FAM107A, IGLL1, SELE and T stage were independent prognostic factors of LUSC (Table 4). The regression model we constructed using multivariate Cox analysis can be used to predict LUSC patient OS. According to the median score, patients were divided between high- and low-risk groups. In training, Kaplan–Meier indicated patient OS in the low-risk group was significantly lower than in the high-risk group, as confirmed by external validation using data from the GEO database (GSE73403) (Fig. 5).

**Table 4:** Cox proportional hazards model analysis of prognostic factors.

Variables	Univariate Cox analysis			Multivariate Cox analysis		
	HR	95% CI	*P*	HR	95% CI	*P*
FAM107A	1.015	1.01–1.025	0.0035	1.0156	1.00–1.03	0.044
IGLL1	1.062	1.01–1.113	0.0124	1.0723	1.02–1.127	0.006
SELE	1.025	0.99–1.054	0.05	1.0006	1.01–1.045	0.012
PIGR	1.011	0.98–1.023	0.542	0.9977	0.99–1.004	0.66
ADH1B	1.022	0.097–1.02	0.548	1.1697	0.99–1.007	0.62
T	1.94	0.95–3.959	0.068	1.8135	0.767–1.78	0.03
M	1.85	0.59–5.841	0.291	1.7957	1.05–3.12	0.47
N	1.156	0.84–1.564	0.379	1.2321	0.84–3.86	0.12

### Development of a nomogram

**Figure 6:**
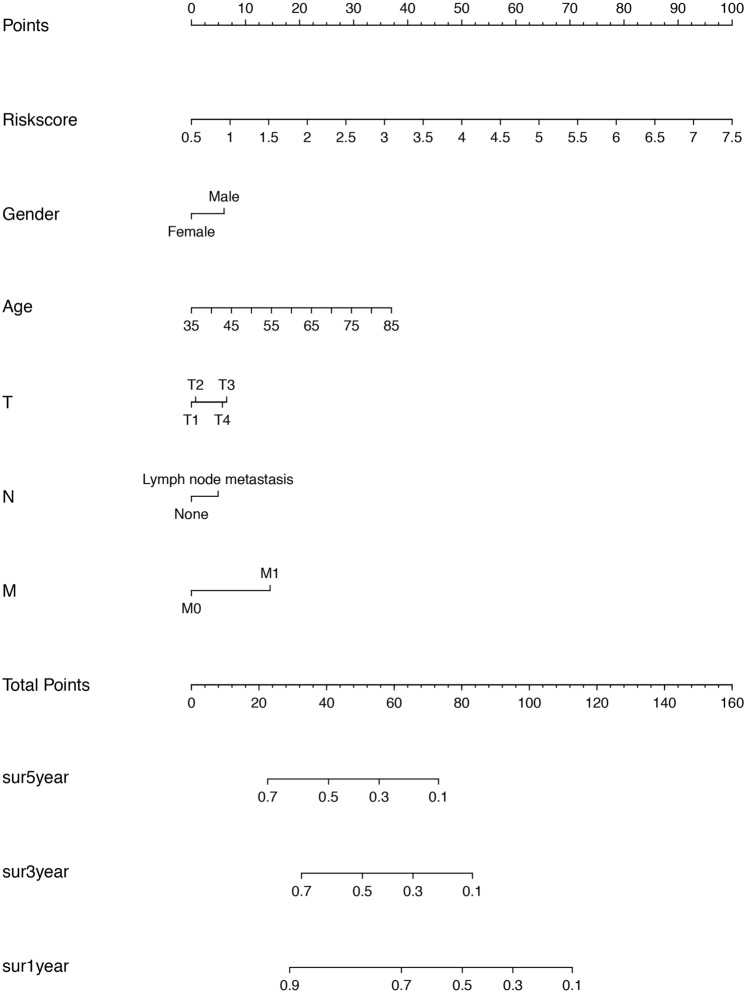
A prognostic nomogram predicting 1-, 2-, and 3-year overall survival of LUSC patient.

A nomogram able to predict LUSC patient survival at 1 year, 3 years, 5 years was constructed, incorporating the following information: T stage, M stage, N stage, age, gender and risk score model. In this nomogram, lines under each independent prognostic factor correspond to a score, with a combined score produced by summing all individual scores. This overall score allows prediction of patient prognosis after 1 years (“sur1year”), 3 years (“sur3year”) and 5 years (“sur5year”) (Fig. 6).

### Nomogram validation

**Figure 7:**
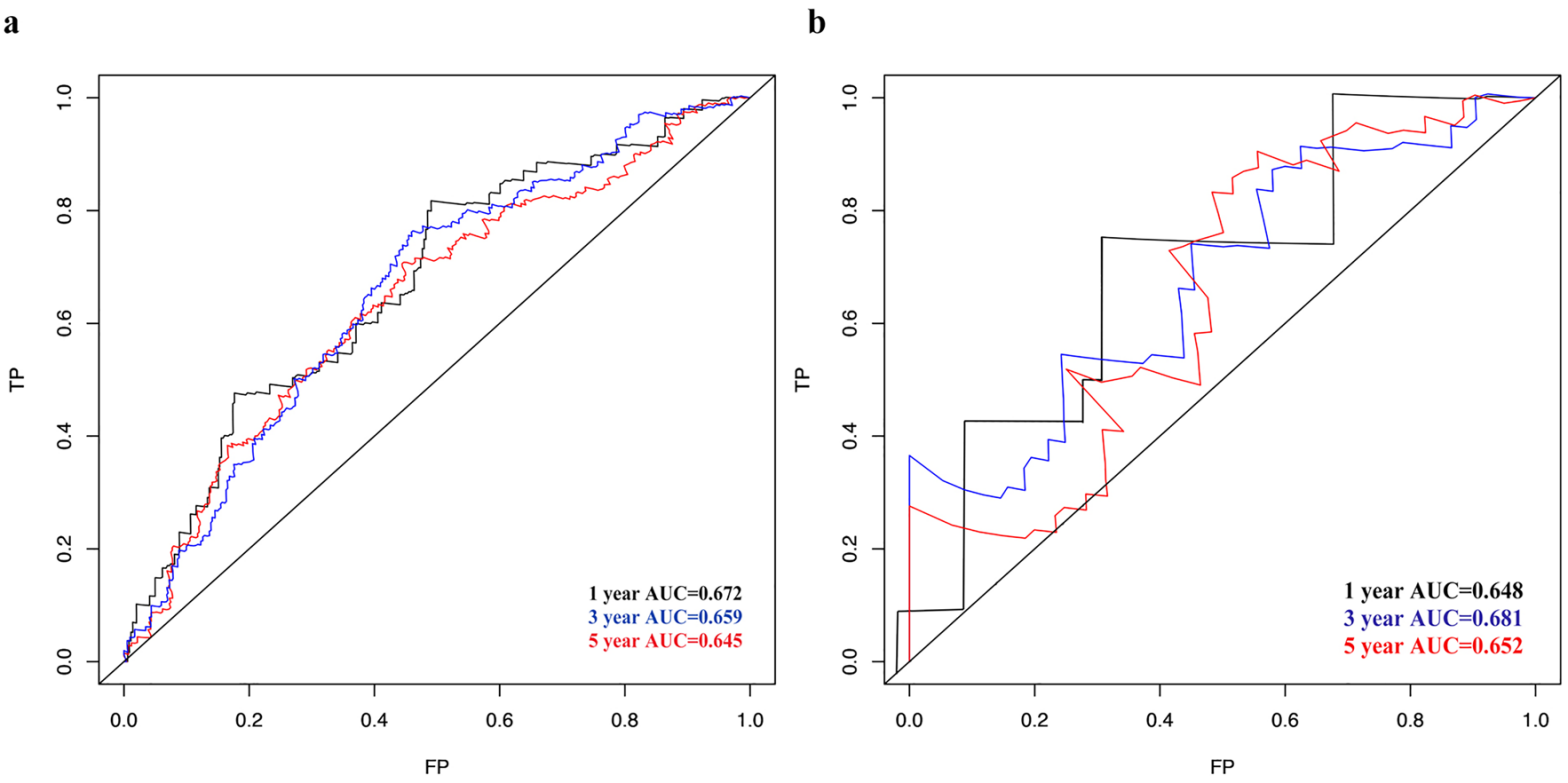
ROC for 1-, 3-, and 5-year overall survival predictions for the nomogram. (**a**) In the training cohort, ROC curve for 1-year, 3-year and 5-year overall survival. (**b**) In the validation cohort, ROC curve for 1-year, 3-year and 5-year overall survival.

We then validated this nomogram using Receiver Operator Characteristic (ROC) curves (Fig. 7). We found that in training the area under the ROC curve was 0.672 (95%CI: 0.632–0.684) for 1-year survival, 0.659 (95%CI: 0.621–0.665) for 3-year survival, and 0.645 (95%CI: 0.621–0.665) for 5-year survival (Fig. 7). In external validation the corresponding areas were 0.648 (95%CI: 0.632–0.684) for 1-year survival, 0.681 (95%CI: 0.661–0.695) for 3-year survival, and 0.652 (95%CI: 0.621–0.665) for 5-year survival.

## Discussion

LUSC is a common type of lung cancer with high incidence and mortality rate. Despite the improved diagnosis and therapy of LUSC, the prognosis is still very poor^11^. As the survival of NSCLC patients is influenced by many factors beyond just tumor stage, the use of the TNM staging system to estimate patient prognosis can often lead to inaccurate survival estimates. Many other studies have generated prognostic models aimed at more accurately estimating the survival of patients with NSCLC in a comprehensive manner^12^.

TMB can alter responses to immunotherapy and affect the prognosis of many cancers, including breast cancer^13^, lung cancer^14^ and colon cancer^15^. We identified 30 genes differentially expressed between high- and low-expression TMB groups. This included 4 up-regulated genes and 26 down-regulated genes. Univariate and multivariate analyses were used to examine the influence of each gene and clinical characteristic on OS, allowing a combined model and nomogram to be developed to predict LUSC patient OS.

GO enrichment analysis indicated these DEGs were mainly involved in lymphocyte activation and lymphocyte/leukocyte/monocyte proliferation. These genes are likely to be closely related to the functioning of immune checkpoint inhibitors in the cancer microenvironment. The proliferation, activation, and differentiation of lymphocytes are critical to the immune system^16^. Many studies have indicated TMB to be an independent predictor of immune checkpoint inhibitor therapy influencing the immune microenvironment^17^. Our work supports the view that TMB is closely related to immunity.

Univariant analysis and multivariate Cox analysis revealed that FAM107A, IGLL1 and SELE were independent prognostic factors of LUSC. These genes reportedly participate in the pathological processes of the TMB microenvironment. FAM107A, a putative tumor suppressor, was originally identified within common missing area of 3p21 in renal cell carcinoma^18^. FAM107A plays a vital role in lung carcinogenesis^19^. Katarzyna Kiwerska et al. found that the expression of FAM107A was reduced significantly in larynx squamous cell carcinoma (LSCC)^20^, and the recurrent inactivation of FAM107A may be involved LSCC development. FAM107A expression was low or even absent in Hodgkin Reed-Sternberg (HRS) cells^21^. IGLL1 is part of the immunoglobulin gene superfamily, and its expression is closely related to humoral immunity^22^. It is associated with immune cell progression^23^. IGLL1 is thought to be a component of pre-BCR (precursor B cell antigen receptor)^24^. IGLL1 Mutations can induce blood system disease^25^. SELE, a selectin, is a cell adhesion molecule that contributes significantly to tumorigenesis and tumor progression^26^. By studying TCGA data, we found that low SELE expression was associated with worse OS in female lung cancer patients who never smoked^27^. Kang et al. found that E-selectin could act as a circulating signaling molecule and facilitate tumor progression and metastasis^28^. SELE was associated with several cancers, including lung cancer^29^, prostate cancer^30^ and colon cancer^31^.

The PPI network indicated that CD22, TLR10, PIGR, and SELE were hub genes. CD22, a transmembrane glycoprotein, is thought to be a regulator of autoimmunity and B cell responses^32,33^. It is also expressed in human lung tumors^34,35^. CD22 is an important drug target for ameliorating autoimmune diseases and acute lymphoblastic leukemia^36,37^. TLR-10 is a pattern recognition receptor with anti-inflammatory properties^38^. Kopp et al. found that TLRs are associated with colorectal cancer via nuclear transcription factor-κB-initiated transcription of inflammatory genes^39^. TLR-10 is particularly important in asthma genetics^40^. It is an anti-inflammatory factor affecting the risk of tuberculosis^41^. Polymeric immunoglobulin receptor (PIGR) is a component of the mucosal immune system correlated with several cancers, such as pancreatic cancer^42^, colon cancer^43^, hepatocellular carcinoma^44^ and bladder cancer^45^.

TMB has a clear role in tumorigenesis and development. It is associated with the immune microenvironment and inflammation. In this study, using TMB-associated genes we developed a three gene risk model to predict patient survival. Patient mortality increases with increasing risk score. We also constructed a nomogram based on the TMB-associated genes and clinical characteristic to predict LUSD patient OS.

This study has shortcomings. First, there is no direct experimental verification of the identified DEG in LUSC. Secondly, there is not a large enough number of clinical samples to confirm the risk model and nomogram. Larger clinical trials will be needed to verify this in the future.

## Conclusion

TMB correlated with tumourigenesis and the development of LUSC. We constructed a TMB-associated risk score and nomogram to predict LUSC patient survival.
